# Itemanalyse der Kurzformen des Tinnitusfragebogens: Mini-TF-12 und Mini-TF-15

**DOI:** 10.1007/s00106-023-01365-z

**Published:** 2023-09-13

**Authors:** Petra Brueggemann, Gerhard Goebel, Benjamin Boecking, Nienke Hofrichter, Matthias Rose, Birgit Mazurek

**Affiliations:** 1 Tinnituszentrum, Charité – Universitätsmedizin Berlin, Luisenstraße 13, 10117 Berlin, Deutschland; 2 Klinik für Innere Medizin und Psychosomatik, Charité – Universitätsmedizin Berlin, Berlin, Deutschland; 3 Tinnitus- und Hyperakusis-Zentrum, Neurozentrum Prien, Prien am Chiemsee, Deutschland

**Keywords:** Lebensqualität, Erhebungen und Fragebögen, Psychischer Stress, Reliabilität und Validität, Sensitivität und Spezifität, Quality of life, Surveys and questionnaires, Psychological distress, Reliability and validity, Sensitivity and specificity

## Abstract

**Hintergrund:**

Eine kurze, aber zuverlässige Messung des tinnitusbedingten Leidensdrucks ist von entscheidender Bedeutung für das Verständnis und die Therapieoptionen für dieses häufig sehr einschränkende Symptom. Im deutschen Raum werden mehrere Selbstauskunftsfragebögen benutzt, und für die deutsche Übersetzung des Tinnitusfragebogens (TF) existieren mehrere Kurzvarianten.

**Zielsetzung:**

In der vorliegenden Arbeit sollten der häufig benutze Mini-TF-12-Fragebogen und der neu entwickelte Mini-TF-15-Fragebogen hinsichtlich Reliabilität, Itemschwierigkeit, Sensitivität (Trennschärfe) und Vorhersagekraft der Items untersucht werden, um eine Entscheidung für den einen oder anderen Fragebogen in verschiedenen Untersuchungskontexten zu erleichtern.

**Methoden:**

Die Daten von 1409 Patienten mit chronischem Tinnitus, die die deutsche Version des 52-Item-TF und zusätzliche psychologische Tests (Allgemeine Depressionsskala – Langform, ADS‑L; Berliner Stimmungsfragebogen, BSF; Patient Health Questionnaire, PHQ; Anamnestic Comparative Self-Assessment, ACSA, und Fragebogen zu Selbstwirksamkeit – Optimismus – Pessimismus, SWOP) im Tinnituszentrum der Charité – Universitätsmedizin Berlin ausfüllten, wurden retrospektiv analysiert. Dazu wurde eine externe Validierung der Items verschiedener Versionen des TF durchgeführt (deutsche Originalversion TF, Mini-TF-12 und Mini-TF-15).

**Ergebnisse:**

Die Items des Mini-TF-12 und des Mini-TF-15 korrelierten spezifisch mit der Gesamtskala. Beide Kurzfragebögen sind hinsichtlich Reliabilität und Itemkonstruktion (Schwierigkeit, Trennschärfe) von vergleichbarer Güte.

**Schlussfolgerung:**

Beide Fragebögen weisen eine sehr gute Itemqualität auf und sind klinisch effizienter als die Langform des TF einzusetzen. Sollte jedoch für Forschungsfragen eine Ähnlichkeit der Faktorstruktur zum Originalfragebogen gefordert sein, empfiehlt sich der Einsatz des Mini-TF-15.

## Abstract (en)

**Background:**

A brief but reliable measurement of tinnitus-related distress is crucial for understanding and treatment options for this often very limiting symptom. Several self-report questionnaires are used in German-speaking countries and several short versions exist for the German translation of the Tinnitus Questionnaire (TQ).

**Objective:**

In the present work, the frequently used Mini-TQ-12 questionnaire and the newly developed Mini-TQ-15 questionnaire will be investigated with regard to reliability, item difficulty, sensitivity (discriminatory power), and predictive power of the items in order to facilitate a decision for one or the other questionnaire in different examination contexts.

**Methods:**

Data from 1409 patients with chronic tinnitus who completed the German 52-item version of the TQ and additional psychological tests (ADS‑L, BSF, PHQ, ACSA and SWOP) at the Tinnitus Centre of the Charité Universitaetsmedizin Berlin, Germany, were retrospectively analyzed. We performed external validation of items from different versions of the TQ (original version, Mini-TQ-12, and Mini-TQ-15).

**Results:**

The items of the Mini-TQ-12 and the Mini-TQ-15 predicted specifically the total score. Both short questionnaires are of comparable quality in terms of reliability and item construction (difficulty, discriminatory power).

**Conclusion:**

Both questionnaires have a very good item quality and are clinically more efficient to use than the long-form TQ. If a similarity of the factor structure to the original questionnaire is required for research questions, the use of the Mini-TQ-15 is recommended.

Tinnitus ist definiert als das Auftreten einer Phantomschallwahrnehmung in Abwesenheit einer entsprechenden externen Schallquelle, und aktuell wird unabhängig davon eine Tinnitusstörung über das damit verbundene Belastungsempfinden definiert [13]. Die Tinnitusprävalenz in der erwachsenen Bevölkerung beträgt berechnet über verschiedene Studien weltweit 14,4 %, die Prävalenz für chronischen Tinnitus wird dabei mit 9,8 % angegeben, bei 3,4 % der Bevölkerung wird das Ohrgeräusch nach Vorstellung bei einem Mediziner durch Vergabe einer entsprechenden klinischen Diagnose relevant [29]. Die Prävalenz steigt – ebenso wie die Schwere des Tinnitus – typischerweise mit dem Alter, was u. a. mit der deutlichen Zunahme von Schwerhörigkeit im höheren Lebensalter in Zusammenhang gebracht wird [3, 9, 11, 39]. Bei 1,2 % der Bevölkerung bestehen aufgrund des Tinnitus starke Belastungen und Beeinträchtigungen in verschiedenen Lebensbereichen [4]. An Tinnitus erkrankte Patienten leiden zudem häufig unter psychischen Komorbiditäten, wie Depression, Angst und somatoformen Störungen [22, 37, 42]. Umgekehrt gilt, dass insbesondere Patienten, die durch den Tinnitus stark belastet sind, häufiger und stärker psychische Beschwerden entwickeln [32, 43]. Die Erfassung und Therapie tinnitusassoziierter Beschwerden sollte daher stets unter besonderer Berücksichtigung der mit dem Tinnitus assoziierten psychologischen Aspekte erfolgen.

Dafür kommen Selbstbewertungsfragebögen wie der Tinnitusfragebogen TF [21] zum Einsatz, der international in der klinischen Routine verwendet wird. Aufgrund der hohen Heterogenität von chronischem Tinnitus in Bezug auf Schwere, Art der Beeinträchtigungen sowie somatischen und psychischen Komorbiditäten [12] wird zusätzlich zur Erfassung der Gesamtbelastung eine differenzierte Betrachtung von Beschwerden in verschiedenen Bereichen (Domänen) angestrebt. Diese modulare Erfassung von Beschwerden erlaubt eine genauere Untersuchung des Gesundheitserlebens von Patienten mit chronischem Tinnitus.

Bereits Hallam et al. [21] untersuchten mittels Faktorenanalyse verschiedene Domänen tinnitusassoziierter Beschwerden für die englischsprachige Originalversion des TF (TQ). Hiller und Goebel [24] führten ähnliche Untersuchungen für die deutschsprachige Version durch. In der englischsprachigen Originalversion des TF (TQ) wurden dabei 3 Hauptfaktoren identifiziert:

- emotionale Belastung („emotional distress“),
- Hörprobleme („auditory perceptual difficulties“) und
- Schlafstörungen („sleep disturbances“).

Hiller und Goebel [24] beschrieben hingegen 5 Domänen:

- emotionale und kognitive Belastung,
- Penetranz („intrusiveness“),
- Hörprobleme,
- Schlafstörungen und
- körperliche Beschwerden.

In neueren Studien wurde die Kriteriumsvalidität des TF mit den deutschsprachigen Versionen des Tinnitus Handicap Inventory (THI) und Tinnitus Functional Index (TFI) verglichen [5]. Die Gesamtscores der 3 Fragebögen zeigen eine hohe konvergente Validität und damit eine Vergleichbarkeit über klinische und Forschungskontexte hinweg. Im Gegensatz dazu weisen aber die Ergebnisse der Subskalen hohe Inkonsistenz auf. Es wird kritisch diskutiert, inwieweit die Subskalen des TF in eine Auswertung einbezogen werden sollten [19, 20].

Da die Originalversion des TF mit 52 Items zudem relativ umfangreich und zeitintensiv in der Beantwortung ist, was von Patienten mitunter als belastend erlebt wird und dazu führen kann, dass der Fragebogen unvollständig ausgefüllt wird, stellen Kurzversionen des TF geeignete Alternativen zur schnelleren und wirtschaftlicheren Erfassung tinnitusassoziierter Beschwerden dar. Hiller und Goebel [25] entwickelten hierfür eine 12 Items umfassende Kurzversion (Mini-TF). Frühere Studien zeigten hinsichtlich der Faktorenstruktur des Mini-TF jedoch teils widersprüchliche Ergebnisse mit ein- [25], zwei- [38] oder dreifaktoriellen Strukturen [40]. Diese Ergebnisse deuten darauf hin, dass der Mini-TF keine ähnlich differenzierte, domänenspezifische Erfassung tinnitusassoziierter Beschwerden erlaubt wie der ursprüngliche englischsprachige TF, da er keine der Originalversion vergleichbare Faktorstruktur aufweist.

Daher wurde eine neue Kurzversion (Mini-TF-15) entwickelt [26]. Diese wies eine dreifaktorielle Struktur auf und enthielt je 5 Items, die sich auf die 3 Faktoren emotionale Belastung, Hörprobleme und Penetranz des TF bezogen. Mittels ROC-Analyse wurde zudem ein Grenzwert von 16 Punkten für den Mini-TF-15 ermittelt. Somit kann der Mini-TF-15 wie auch der Mini-TF-12 zur klinisch bedeutsamen Differenzierung zwischen Patienten mit kompensiertem und dekompensiertem Tinnitus eingesetzt werden. Die Konstruktvalidität der beiden Kurzversionen wurde in einer nachfolgenden Studie für TF, Mini-TF-12 und Mini-TF-15 auf der Dimension „Depressivität“ als konstruktnahes Merkmal berechnet [27]. Dabei wurde untersucht, inwieweit die einzelnen Faktoren des TF, Mini-TF-12 und Mini-TF-15 mit den Ergebnissen anderer Fragebögen korrelieren, die zur Messung des gleichen Merkmals zum Einsatz kommen (Konstruktvalidität). Dafür wurde die Korrelation zwischen dem Gesamtscore der Allgemeinen Depressionsskala – Langform (ADS-L) [15] und dem Faktor emotionale Belastung des TF berechnet, dessen Items sich ebenfalls auf Depressionssymptome wie Niedergeschlagenheit, Hoffnungslosigkeit und pessimistische Zukunftserwartungen beziehen. Diese Korrelation wurde mit den Korrelationen verglichen, die zwischen den 5 Faktoren des TF bestanden und die möglichst voneinander diskriminante Aussagen ergeben sollten. Die 5 Faktoren des TF entstammen dem gleichen Fragebogen, werden jedoch zur Messung verschiedener Merkmale (Diskriminanzvalidität) verwendet [27].

Die externe Validierung der Faktorenstruktur des Mini-TF-15 zeigte hohe, spezifische Korrelationen zwischen dem Faktor emotionale Belastung des Mini-TF-15 und den Depressionsskalen ADS‑L, Patient Health Questionnaire (PHQ-9) [33, 34] und BSF(Berliner Stimmungsfragebogen)-Ängstliche Depressivität [28]. Im Fall des Mini-TF-12 bestanden dahingegen unspezifisch hohe Korrelationen zwischen den Depressionsfragebögen und beiden Faktoren, die zudem eine hohe Korrelation untereinander zeigten. Insgesamt erlaubt der Mini-TF-12 somit im Unterschied zum Mini-TF-15 keine differenzierte Erfassung tinnitusassoziierter Beschwerden in spezifischen Domänen.

Zudem waren die Korrelationen zwischen den 3 Faktoren des Mini-TF-15 niedriger als zwischen dem Faktor emotionale Belastung des Mini-TF-15 und den 3 Depressionsskalen. Dies weist insgesamt auf eine gute Konstrukt- und Diskriminanzvalidität hin. Gleichzeitig waren Korrelationen zwischen den Faktoren emotionale Belastung und Penetranz sowohl im Fall des TF als auch beim Mini-TF-15 vergleichsweise hoch. Dies deutet darauf hin, dass durch beide Faktoren möglicherweise ähnliche bzw. sich teilweise überlappende Konstrukte gemessen werden.

Somit ist eine generelle Untersuchung der Kriteriums- und Konstruktvalidität der beiden Fragebögen Mini-TF-12 (Mini-TF umbenannt, zu besseren Unterscheidung von Mini-TF-15) und Mini-TF-15 bereits erfolgt. In der vorliegenden Studie wurden die Itemcharakteristika von Mini-TF-12 und Mini-TF-15 verglichen, um Hinweise auf ihre klinische Anwendbarkeit zu geben.

Dafür wurden folgende Berechnungen durchgeführt:

- Die Itemschwierigkeit des Mini-TF-15 wurde mit der des Mini-TF-12 verglichen.
- Vergleich der Trennschärfe der Items zwischen Mini-TF-12 und Mini-TF-15.
- Die Reliabilität der beiden Kurzfragebögen wurde berechnet.
- Die Vorhersagekraft der Einzelitems von Mini-TF-12 und Mini-TF-15 für den Gesamtscore wurde jeweils über lineare Regression dargestellt.

## Methodik

### Konstruktion der Fragebögen zur Untersuchung des chronischen Tinnitus

Der *Tinnitusfragebogen, *TF [16, 21, 24], stellt ein Instrument zur Beurteilung des Gesundheitserlebens von Patienten mit chronischem Tinnitus aus Patientensicht dar. Patientenzentrierte Instrumente zur Messung des subjektiv empfundenen Gesundheitszustands und Belastungserlebens haben in der Behandlung von Patienten mit chronischen Erkrankungen zunehmend an Bedeutung gewonnen [7]. Aktuell sind für den deutschsprachigen Raum mehrere Fragebögen zur Erfassung tinnituskorrelierter Beschwerden validiert [5]. Der TF hat sich in vielen Studien als reliabel und valide bewährt [1]. Hallam et al. [21] entwickelten die englischsprachige Originalversion des TF. Dieser enthält 52 Aussagen (Items) zu tinnitusbezogenen Beschwerden, wie beispielsweise Item 17: „Wenn die Ohrgeräusche andauern, wird mein Leben nicht mehr lebenswert sein.“ Für jede Aussage vergibt der Patient 2 Punkte („stimmt“), 1 Punkt („stimmt teilweise“) oder 0 Punkte („stimmt nicht“). Der Gesamtscore dient als Maß für die Schwere tinnitusassoziierter Beschwerden und der damit verbundenen Gesamtbelastung. Ein Gesamtscore von ≤ 46 Punkten gilt als kompensierter Tinnitus, bei einem Summenwert von ≥ 47 Punkten ist dagegen von einem dekompensierten Tinnitus mit hoher Belastung und relevanten Beeinträchtigungen auszugehen. Die deutschsprachige Version des TF hat 52 Items [16, 24, 25]. Dieser Fragebogen war lange Zeit das einzige für den deutschen Sprachraum adaptierte Selbstbeurteilungsinstrument für Tinnitusbelastung und fand klinisch entsprechend weite Verbreitung.

Bereits Hallam et al. [21] führten ebenso wie Hiller und Goebel [24] faktorenanalytische Untersuchungen durch, um den 52 Items des TF zugrunde liegende Faktoren zu ermitteln und so das Belastungserleben bei chronischem Tinnitus aus Patientensicht differenzierter und systematischer erfassen zu können. Dabei wurden folgende 3 Faktoren in ähnlicher Weise sowohl von Hallam et al. [21] als auch von Hiller und Goebel [24] identifiziert: emotionale Belastung („emotional distress“), Hörprobleme („auditory perceptual difficulties“) und Schlafstörungen („sleep disturbances“).

Für die verkürzte Form des Fragebogens, *Mini-TF-12* (Mini-TF) [25], wurden aus der deutschsprachigen Langform des TF Items mit guter Reliabilität auf Itemniveau ausgewählt. Die Test-Retest-Reliabilität betrug 0,89, das Cronbach‑α wurde im Sinne guter Werte für interne Konsistenz mit 0,87‑0,9 bewertet. Die Ergebnisse für die Subskalen korrelierten unbefriedigend mit der Langform des Fragebogens. Dies gilt besonders für Hörprobleme und Schlafstörungen. Mehrere konfirmatorische Faktoranalysen (CFA) zur Überprüfung der Fakorstruktur des Mini-TF [25] führten zu unterschiedlichen Ergebnissen. An verschiedenen Stichproben wurde eine ein- [25], zwei- [38] oder dreifaktorielle [40] Struktur berechnet mit in Großem und Ganzen befriedigenden Ergebnissen (einfaktoriell: CFI = 0,870; TLI = 0,841; RMSEA = 0,092; zweifaktoriell: CFI = 0,894; TLI = 0,868; RMSEA = 0,090; dreifaktoriell: CFI = 0,890; TLI = 0,858; RMSEA = 0,093), aber damit ungenügender Vergleichbarkeit. Der Mini-TF-12 erwies sich als sensitiv für Veränderungen durch Therapie, und als Schwellenwerte wurden 8‑12 Punkte als moderate Tinnitusbelastung, 13–18 Punkte als schwere Tinnitusbelastung und 19–24 Punkte als sehr schwere Tinnitusbelastung vorgeschlagen.

**Figure 1:**
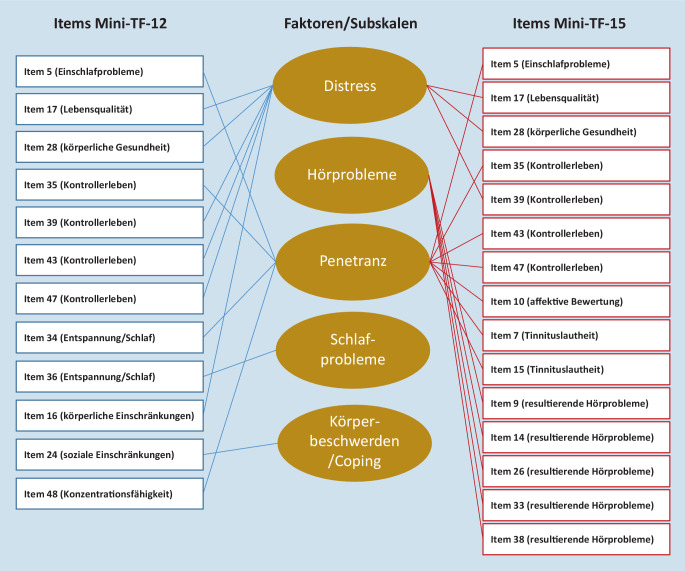

Nachdem faktorenanalytische Untersuchungen des Mini-TF-12 3 unterschiedliche Faktorenmodelle [25, 38, 40] und damit nicht mit der Langform des TF vergleichbare Ergebnisse gezeigt hatten, wurde eine neue, 15 Items umfassende Kurzversion, *Mini-TF-15*, entwickelt [26]. Hierfür wurden für jeden der 3 Faktoren emotionale Belastung, Hörprobleme und Penetranz des TF diejenigen 5 Items ausgewählt, die die höchsten Ladungskoeffizienten in der CFA gezeigt hatten. Zur besseren Vergleichbarkeit der beiden Kurzformen wurden Items, die auch im Mini-TF-12 enthalten sind, bevorzugt verwendet. Die Ergebnisse einer weiteren exploratorischen (EFA) und konfirmatorischen Faktoranalyse (CFA) stützten die vermutete dreifaktorielle Struktur des Mini-TF-15, und die Items des resultierenden Mini-TF-15 im Vergleich zum Mini-TF-12 sind in Abb. 1 dargestellt.

Receiver-Operating-Characteristic(ROC)-Analysen (dekompensiert vs. kompensiert an der Langform des TF operationalisiert) ergaben zudem einen Cut-off-Wert von 16 Punkten für kompensierte vs. für den Mini-TF-15.

Die 12 Items der deutschsprachigen Version des Mini-TF-12 und die 15 Items des Mini-TF-15 sind in Tab. 1 und 2 aufgeführt.

### Stichprobenbeschreibung

Für die Konstruktion des Mini-TF-12 wurden 2 verschiedene Stichproben genutzt: 351 stationäre psychosomatische Patienten aus der Roseneck-Klinik in Prien (Durchschnittsalter 47,4 Jahre; 31,8 % weiblich und 68,2 % männlich) und 122 ambulante Patienten aus einer psychologischen Institutsambulanz der Universität Mainz (Durchschnittsalter 49,8; 43,4 % weiblich und 56,6 % männlich) [25].

Für die Konstruktion des Mini-TF-15 wurden die Ergebnisse der deutschsprachigen 52-Items umfassenden Originalversion des TF [24] in einer großen Stichprobe (*n* = 7112) von Patienten mit chronischem Tinnitus retrospektiv ausgewertet. Hierbei wurden Testdaten der volljährigen Patienten mit chronischem Tinnitus eingeschlossen, die im Zeitraum vom 06.03.2003 bis zum 11.05.2016 in der Tagesklinik des Tinnituszentrums der Charité – Universitätsmedizin Berlin untersucht worden waren (Ethik-Nr. EA4_137_20). Alle Daten wurden in der klinischen Routine erhoben [26]. Die Gesamtstichprobe (*n* = 7112) setzte sich aus 3496 (49,2 %) weiblichen und 3616 (50,8 %) männlichen Patienten mit chronischem Tinnitus zusammen. Das Durchschnittsalter betrug 50,8 Jahre (Standardabweichung, SA: 13,1; Spannbreite: 18–90 Jahre). Der durchschnittliche Gesamtscore der 52-Item-Version des TF [16] betrug 37,1 (SA = 17,6). Bei 4977 (70 %) der Patienten lag ein kompensierter Tinnitus (TF-Gesamtscore ≤ 46) und bei 2135 (30 %) ein dekompensierter Tinnitus (TF-Gesamtscore ≥ 47) vor.

### Statistische Methoden

Zur statistischen Auswertung kam das Softwareprogramme „Statistical Package for the Social Sciences“ (SPSS, Version 29.0, IBM® SPSS® Statistics) zum Einsatz. Deskriptive Analysen erfolgten zur Untersuchung der Itemschwierigkeit und Trennschärfe. Die Reliabilitätsmessung auf Itemebene erfolgte mit der Split-Half-Methode, und das Cronbach‑α wurde berechnet. Der Vergleich der Itemrelevanz erfolgte über Prädiktionsgewichte im jeweiligen Regressionsmodell (mit Gesamtscore von Mini-TF-12 und Mini-TF-15 als abhängigen Variablen).

## Ergebnisse

### Itemschwierigkeit

Der *Schwierigkeitsindex p* eines Items ist der Quotient aus der bei diesem Item tatsächlich erreichten Punktsumme aller Probanden (*n*) und der maximal erreichbaren Punktsumme, multipliziert mit 100. Als optimaler Schwierigkeitsindex für mehrstufige Skalen wird eine Itemschwierigkeit von 50 % angesehen, wobei Items unter 20 und über 80 % in der Regel ausgeschieden werden.

**Table 1:** 

Item aus Original-TF (Langform)^a^	Gesamtstichprobe (*n* = 7112)			Schwierigkeits-index *p* in %
	M	SD	SK	
34: Wegen der Ohrgeräusche fällt es mir schwer, mich zu entspannen	1,38	0,69	−0,66	69,0
35: Oft sind meine Ohrgeräusche so schlimm, dass ich sie nicht ignorieren kann	1,38	0,69	−0,66	69,0
48: Die Ohrgeräusche haben meine Konzentration beeinträchtigt	1,09	0,75	−0,15	54,5
39: Wegen der Ohrgeräusche bin ich leichter niedergeschlagen	0,92	0,78	0,14	46,0
36: Wegen der Ohrgeräusche brauche ich länger zum Einschlafen	1,07	0,82	−0,12	53,5
24: Aufgrund der Ohrgeräusche bin ich mit meiner Familie und meinen Freunden gereizter	0,80	0,75	0,35	40,0
5: Ich bin mir der Ohrgeräusche vom Aufwachen bis zum Einschlafen bewusst	1,25	0,73	−0,43	62,5
16: Ich mache mir wegen der Ohrgeräusche Sorgen, ob mit meinem Körper ernsthaft etwas nicht in Ordnung ist	0,87	0,79	0,23	43,5
28: Ich sorge mich, dass die Ohrgeräusche meine körperliche Gesundheit schädigen könnten	0,87	0,81	0,24	43,5
43: Ich denke oft darüber nach, ob die Ohrgeräusche jemals weggehen werden	1,20	0,78	−0,37	60,0
17: Wenn die Ohrgeräusche andauern, wird mein Leben nicht lebenswert sein	0,53	0,67	0,88	50,0
47: Ich bin ein Opfer meiner Ohrgeräusche	0,50	0,69	1,04	25,0

*M* Mittelwert, *SD* Standardabweichung, *SK* Schiefe (0 symmetrisch, positiv linksschief, negativ rechtsschief), *TF* Tinnitusfragebogen

^a^Items aus TF, entwickelt von Hallam et al. In der vorliegenden Studie wurde die deutsche Version des TF genutzt

**Table 2:** 

Item aus Original-TF (Langform)^a^	Gesamtstichprobe (*n* = 7112)			Schwierigkeitsindex *p* in %
	M	SD	SK	
47: Ich bin ein Opfer meiner Ohrgeräusche	0,50	0,69	1,04	25,0
17: Wenn die Ohrgeräusche andauern, wird mein Leben nicht lebenswert sein	0,53	0,67	0,88	26,5
43: Ich denke oft darüber nach, ob die Ohrgeräusche jemals weggehen werden	1,20	0,78	−0,37	60,0
39: Wegen der Ohrgeräusche bin ich leichter niedergeschlagen	0,92	0,78	0,14	46,0
28: Ich sorge mich, dass die Ohrgeräusche meine körperliche Gesundheit schädigen könnten	0,87	0,81	0,24	43,5
33: Wegen der Ohrgeräusche ist es für mich schwieriger, einer Unterhaltung zu folgen	0,87	0,78	0,23	43,5
14: Wegen der Ohrgeräusche ist es für mich schwieriger, mehreren Menschen gleichzeitig zuzuhören	1,07	0,85	−0,13	53,5
38: Wegen der Ohrgeräusche fällt es mir schwerer zu telefonieren	0,66	0,77	0,66	33,0
9: Wegen der Ohrgeräusche habe ich Schwierigkeiten zu sagen, woher andere Töne kommen	0,80	0,75	0,34	40,0
26: Aufgrund der Ohrgeräusche erscheinen mir die Stimmen anderer Menschen verzerrt	0,41	0,65	1,31	20,5
5: Ich bin mir der Ohrgeräusche vom Aufwachen bis zum Einschlafen bewusst	1,25	0,73	−0,43	62,5
35: Oft sind meine Ohrgeräusche so schlimm, dass ich sie nicht ignorieren kann	1,38	0,69	−0,66	69,0
15: Die Ohrgeräusche sind die meiste Zeit laut	1,05	0,76	−0,09	52,5
7: Meistens sind die Ohrgeräusche ziemlich leise	0,76	0,72	0,40	38,0
10: Die Art, wie die Ohrgeräusche klingen, ist wirklich unangenehm	1,52	0,61	−0,88	76,0

*M* Mittelwert, *SD* Standardabweichung, *SK* Schiefe (0 symmetrisch, positiv linksschief, negativ rechtsschief),* TF* Tinnitusfragebogen

^a^Items aus TF, entwickelt von Hallam et al. In der vorliegenden Studie wurde die deutsche Version des TF genutzt

Die Ergebnisse zeigen, dass die Itemschwierigkeiten sowohl des Mini-TF-12 (Tab. 1) als auch des Mini-TF-15 (Tab. 2) die Kriterien erfüllen und in den meisten Fällen gut ausgeprägt sind. Eine optimale Itemschwierigkeit von 50 % (±10 %) weisen 8 der 12 Items beim Mini-TF-12 auf, beim Mini-TF-15 sind es 7 der 15 Items.

### Vergleich der Itemtrennschärfe

Die *Itemtrennschärfe *(*rit*) wurde durch Korrelation des jeweiligen Items mit dem Gesamtwert des dazu gehörigen Kurzfragebogens bestimmt. Der Wert der Trennschärfe (rit) kann zwischen −1 und 1 liegen. Erfasst das Item etwas Ähnliches wie der Gesamttest, ergibt sich ein hoher positiver Trennschärfeindex. Bei einer Trennschärfe nahe 0 hat das Item zu wenig mit den anderen Items des Tests oder der Fragebogenskala gemeinsam. Als untere Grenze wurde ein Wert von r > 0,30 festgelegt. Diesen Grenzwert unterschritt keines der Items des Mini-TF-12 und des Mini-TF-15 (Tab. 3).

**Table 3:** 

Items Mini-TF-12	Itemtrenn-schärfe rit (mit Mini-TF-12 gesamt)	Items Mini-TF-15	Itemtrenn-schärfe rit (mit Mini-TF-15 gesamt)
34: Wegen der Ohrgeräusche fällt es mir schwer, mich zu entspannen	.60	10: Die Art, wie die Ohrgeräusche klingen, ist wirklich unangenehm	.52
35: Oft sind meine Ohrgeräusche so schlimm, dass ich sie nicht ignorieren kann	.60	35: Oft sind meine Ohrgeräusche so schlimm, dass ich Sie nicht ignorieren kann	.59
48: Die Ohrgeräusche haben meine Konzentration beeinträchtigt	.62	33: Wegen der Ohrgeräusche ist es für mich schwieriger, einer Unterhaltung zu folgen	.49
39: Wegen der Ohrgeräusche bin ich leichter niedergeschlagen	.66	39: Wegen der Ohrgeräusche bin ich leichter niedergeschlagen	.66
36: Wegen der Ohrgeräusche brauche ich länger zum Einschlafen	.53	14: Wegen der Ohrgeräusche ist es für mich schwieriger, mehreren Menschen gleichzeitig zuzuhören	.47
24: Aufgrund der Ohrgeräusche bin ich mit meiner Familie und meinen Freunden gereizter	.60	38: Wegen der Ohrgeräusche fällt es mir schwerer zu telefonieren	.49
16: Ich mache mir wegen der Ohrgeräusche Sorgen, ob mit meinem Körper ernsthaft etwas nicht in Ordnung ist	.47	15: Die Ohrgeräusche sind die meiste Zeit laut	.54
28: Ich sorge mich, dass die Ohrgeräusche meine körperliche Gesundheit schädigen könnten	.58	28: Ich sorge mich, dass die Ohrgeräusche meine körperliche Gesundheit schädigen könnten	.58
43: Ich denke oft darüber nach, ob die Ohrgeräusche jemals weggehen werden	.50	43: Ich denke oft darüber nach, ob die Ohrgeräusche jemals weggehen werden	.51
17: Wenn die Ohrgeräusche andauern, wird mein Leben nicht lebenswert sein	.64	17: Wenn die Ohrgeräusche andauern, wird mein Leben nicht lebenswert sein	.64
47: Ich bin ein Opfer meiner Ohrgeräusche	.60	47: Ich bin ein Opfer meiner Ohrgeräusche	.61
5: Ich bin mir der Ohrgeräusche vom Aufwachen bis zum Einschlafen bewusst	.49	5: Ich bin mir der Ohrgeräusche vom Aufwachen bis zum Einschlafen bewusst	.50
		7: Meistens sind die Ohrgeräusche ziemlich leise	.38
		9: Wegen der Ohrgeräusche habe ich Schwierigkeiten zu sagen, woher andere Töne kommen	.46
		26: Aufgrund der Ohrgeräusche erscheinen mir die Stimmen anderer Menschen verzerrt	.43

*TF* Tinnitusfragebogen

Die Abb. 1 zeigt die Items des Mini-TF-12 und Mini-TF-15 relativ zu den Subgruppenbezeichnungen der TF-Langform.

### Reliabilitätsberechnungen

Als Reliabilitätsmaß wird das Cronbach‑α und der Split-Half-Koeffizient verwendet. Hofrichter et al. [26] berechneten das Cronbach‑α für die Faktorstrukturen vom originalen TF, Mini-TF-12 und Mini-TF-15. Die Analysen ergaben hohe Werte von Cronbach‑α für alle extrahierten Faktoren (Faktoren im TF: zwischen 0,74 und 0,89; Mini-TF-12: zwischen 0,79 und 0,81; Mini-TF-15: zwischen 0,79 und 0,85).

Die Split-Half-Koeffizienten für die Items ergeben für beide Kurztests sehr hohe, gute Werte (Tab. 4 und 5).

**Table 4:** 

Cronbach‑α	Teil 1	Wert	1,000
		Anzahl der Items	6^a^
	Teil 2	Wert	1,000
		Anzahl der Items	6^b^
	Gesamtzahl der Items		12
Korrelation zwischen Formen			1,000
Spearman-Brown-Koeffizient	Gleiche Länge		1,000
	Ungleiche Länge		1,000
Guttman-Split-Half-Koeffizient			0,992

*TF* Tinnitusfragebogen

^a^Die Items sind: TF_TIN34, TF_TIN35, TF_TIN48, TF_TIN39, TF_TIN36, TF_TIN16

^b^Die Items sind: TF_TIN28, TF_TIN43, TF_TIN17, TF_TIN47, TF_TIN05, TF_TIN24

**Table 5:** 

Cronbach‑α	Teil 1	Wert	1,000
		Anzahl der Items	8^a^
	Teil 2	Wert	1,000
		Anzahl der Items	7^b^
	Gesamtzahl der Items		15
Korrelation zwischen Formen			1,000
Spearman-Brown-Koeffizient	Gleiche Länge		1,000
	Ungleiche Länge		1,000
Guttman-Split-Half-Koeffizient			0,996

*TF* Tinnitusfragebogen

^a^Die Items sind: TF_36_TIN10, TF_36_TIN35, TF_36_TIN33, TF_36_TIN39, TF_36_TIN14, TF_36_TIN38, TF_36_TIN15, TF_36_TIN28

^b^Die Items sind: TF_36_TIN43, TF_36_TIN17, TF_36_TIN47, TF_36_TIN05, TF_36_TIN07_umgepolt, TF_36_TIN09, TF_36_TIN26

### Itemgewichte (Regressionsmodell)

Wie aus Tab. 3 und Abb. 1 ersichtlich ist, sind 7 Items aus dem Tinnitusfragebogen – Langform (TF) sowohl im Mini-TF-12 als auch im Mini-TF-15 enthalten: Item 5 (Einschlafprobleme), Item 17 (Lebensqualität), Item 28 (körperliche Gesundheit), Items 35, 39, 43 und 47 (Kontrollerleben). Im Mini-TF-12 kommen dazu noch Items zur Entspannungsfähigkeit/Schlaf (34, 36), zu körperlichen (16) und sozialen (24) Einschränkungen sowie zur Konzentrationsfähigkeit (48). Im Mini-TF-15 kommen zu den 7 gemeinsamen Items aus dem Itempool der Langform (TF) hinzu: Items zur affektiven Bewertung (10) und zur empfundenen Lautheit des Tinnitustons (7, 15), sowie Items zu resultierenden Hörproblemen (9, 14, 26, 33, 38). Im Regressionsmodell (abhängige Variable Gesamtwert des Kurzfragebogens, unabhängige Variable die jeweiligen Items) soll für den Mini-TF-12 (Tab. 6) und den Mini-TF-15 (Tab. 7) gezeigt werden, welches Gewicht (β‑Koeffizient als standardisierte Regressionskoeffizienten) die einzelnen Items für die Vorhersage des Gesamtwerts haben.

**Table 6:** 

Modell		Nicht standardisierte Koeffizienten		Standardisierte Koeffizienten	Signifikanz
		Regressionskoeffizient B	Standardfehler	β‑Koeffizient	
1	(Konstante)	−1,059E-13	0,000	–	1,000
	TIN47	1,000	0,000	0,120	0,000
	TIN17	1,000	0,000	0,116	0,000
	TIN43	1,000	0,000	0,135	0,000
	TIN39	1,000	0,000	0,134	0,000
	TIN28	1,000	0,000	0,139	0,000
	TIN16	1,000	0,000	0,138	0,000
	TIN05	1,000	0,000	0,126	0,000
	TIN35	1,000	0,000	0,120	0,000
	TIN34	1,000	0,000	0,120	0,000
	TIN48	1,000	0,000	0,130	0,000
	TIN36	1,000	0,000	0,142	0,000
	TIN24	1,000	0,000	0,130	0,000

*TF* Tinnitusfragebogen

Für den Mini-TF-12 zeigen sich wie erwartet untereinander recht vergleichbare Itemgewichte mit der stärksten Vorhersage des Gesamtscores durch Item 36 (Schlaf) und der relativ niedrigsten Vorhersage durch Item 17 (Lebensqualität).

**Table 7:** 

Modell		Nicht standardisierte Koeffizienten		Standardisierte Koeffizienten	Signifikanz
		Regressionskoeffizient B	Standardfehler	β‑Koeffizient	
1	(Konstante)	1,343E-13	0,000	–	1,000
	TIN47	1,000	0,000	0,116	0,000
	TIN17	1,000	0,000	0,112	0,000
	TIN28	1,000	0,000	0,134	0,000
	TIN33	1,000	0,000	0,131	0,000
	TIN14	1,000	0,000	0,143	0,000
	TIN38	1,000	0,000	0,129	0,000
	TIN09	1,000	0,000	0,125	0,000
	TIN26	1,000	0,000	0,108	0,000
	TIN15	1,000	0,000	0,127	0,000
	TIN07	1,000	0,000	0,121	0,000
	TIN05	1,000	0,000	0,121	0,000
	TIN10	1,000	0,000	0,101	0,000
	TIN35	1,000	0,000	0,115	0,000
	TIN39	1,000	0,000	0,130	0,000
	TIN43	1,000	0,000	0,130	0,000

*TF* Tinnitusfragebogen

Für den Mini-TF-15 gilt eine vergleichbar gute Vorhersagewichtung der 15 Items für den Gesamtscore wie für den Mini-TF-12. Die stärkste Vorhersage des Gesamtscores erfolgt durch Item 14 (Hörprobleme) und die relativ niedrigste Vorhersage durch Item 10 (affektive Bewertung Tinnituston). Entsprechend dem Vorgehen bei der Konstruktion der Kurzfragebögen zeigen beide Regressionsmodelle keine bedeutsamen Unterschiede bezüglich der Schätzung des Gesamtscores mit ausgeglichenen Anteilen der Einzelitems.

## Diskussion

In der vorliegenden Arbeit wurden die beiden Kurzfragebögen Mini-TF-12 und Mini-TF-15 zum Belastungserleben von Patienten mit chronischem Tinnitus hinsichtlich Itemcharakteristika und Reliabilität verglichen.

Die Überprüfung der Kriteriums- und Konstruktvalidität des Mini-TF-12 [25] und der neuen, 15 Items umfassenden Kurzversion Mini-TF-15 [26] erfolgte bereits [27]. Hierbei ergaben sich im direkten Vergleich in einer randomisierten Population zufriedenstellende Gütekriterien für beide Kurzformen [27]. Allerdings zeigte die Untersuchung der Kurzversion Mini-TF-12 keine mit der Langfassung des TF vergleichbare Faktorenstruktur. Es wurden 2 Faktoren extrahiert, die sich keinem der 5 Faktoren des ursprünglichen TF eindeutig zuordnen ließen. Außerdem war keine eindeutige inhaltliche Unterscheidung zwischen beiden Faktoren möglich, da beide Faktoren Items enthielten, die sich auf depressive Symptome und pessimistische Zukunftserwartungen bezogen. Die Korrelation zwischen beiden Faktoren war zudem mit 0,68 als hoch anzusehen. Der Mini-TF-12 leistet über eine Quartilseinteilung eine Einteilung in Schwergerade der Tinnitusbelastung (mit einem Grenzwert von 0–12 leichtgradiger, 8–12 mittelgradiger, 13–18 schwergradiger und 19–24 schwerstgradiger Belastung) [23].

Die neu entwickelte Kurzversion (Mini-TF-15) wies eine dreifaktorielle Struktur auf, umfasste 15 Items, von denen 5 jeweils auf die 3 Faktoren „emotionale Belastung“, „Hörprobleme“ und „Penetranz des Tinnitus“ luden. Auch der Mini-TF-15 kann zur klinisch bedeutsamen Differenzierung zwischen Patienten mit kompensiertem und dekompensiertem Tinnitus eingesetzt werden (Grenzwert 16).

Um die beiden Kurzformen hinsichtlich ihrer klinischen Anwendung besser differenzieren zu können, wurden in der vorliegenden Studie die 12 bzw. 15 Items der Fragebögen hinsichtlich Itemschwierigkeit, Trennschärfe und Reliabilität genauer untersucht.

Hinsichtlich der Itemschwierigkeit, der Trennschärfe und der Reliabilität ergab sich bei in dieser Stichprobe sehr zufriedenstellenden Gütekriterien für beide Kurzformen kein Unterschied. Beide Kurzfragebögen entsprechen bezüglich der Konstruktion und der Gütekriterien allen Anforderungen an psychologische Selbstbeurteilungsinstrumente und sind damit zuverlässig sowie statistisch gültig. Damit sind sie besonders bei rein klinischen Fragen oder großen Kohorten der Langform des Tinnitusfragebogens (TF) vorzuziehen, da sie hinsichtlich Effizienz und Belastung der Patienten infolge der weit geringeren Itemanzahl und damit deutlich kürzerer Bearbeitungszeit von Vorteil sind.

Bei neu zu planenden Studien für die Forschung sollten unbedingt auch international anerkannte und mittlerweile für den deutschen Sprachraum validierte Fragebögen wie der THI mit 25 Items [10] oder der TFI mit 25 Items [41] wegen einer besseren internationalen Vergleichbarkeit in Erwägung gezogen werden. Für beide und für die Langform des TF konnte im unmittelbaren Vergleich ein Ansprechen auf eine Kurzzeitbehandlung bei einer deutschsprachigen Patientenpopulation mit chronischem Tinnitus gezeigt werden [5]. Dabei scheinen für Patienten mit einem erhöhten Angstniveau oder mehr Stress der TF und der THI besser einsetzbar, wobei der THI aufgrund weniger Items v. a. unter Zeitdruck sinnvoll eingesetzt werden kann.

Bei der deskriptiven Analyse der Items der beiden Kurzversionen, den Regressionsmodellen und der Überprüfung der Konstruktvalidität [27] fällt die etwas breitere Aufstellung des Mini-TF-15 hinsichtlich der zugrunde liegenden Faktorenstruktur auf. Dabei ist allerdings zu beachten, dass die von Hiller und Goebel [24] beschriebene fünffaktorielle Struktur der deutschsprachigen Version des TF nicht mit der dreifaktoriellen Struktur des englischen Originalfragebogens [21] übereinstimmt. Da die Faktoren auch den Subskalen entsprechen, wird aktuell empfohlen, die Subskalen der deutschen Langform des TF nicht mehr zu nutzen, sondern immer nur den Gesamtscore auszuwerten [5, 18]. Während der Mini-TF-12 je nach Untersuchungspopulation eine Ein- bis Zweifaktorlösung (depressive Symptome, nicht gut abgrenzbar Pessimismus) ergibt, wurde in der Population für die Konstruktion des Mini-TF-15 eine Dreifaktorlösung (emotionale Belastung, Hörprobleme und Penetranz) ermittelt [26]. Grundsätzlich sollte in Kenntnis dieser Problematik von beiden Kurzformen nur der Gesamtwert interpretiert werden. Damit gilt die Beschränkung der Ergebnisinterpretation auf den Gesamtscore für die Langform und beide Kurzformen.

Entsprechend ergeben sich in der Regression der Items mit Vorhersagegewichten für den Gesamtwert leichte Unterschiede in der inhaltlichen Gewichtung. Der Mini-TF-12 scheint insgesamt auf psychologische Beeinträchtigung bei Tinnitus gut anzusprechen. Hierbei ist besonders zu erwähnen, dass der Mini-TF-12 noch wie in der Langform (Item 36) ein klinisch relevantes Item zu resultierenden Schlafstörungen enthält, das bei der an der Faktorenstruktur orientierten Konstruktion des Mini-TF-15 entfiel [26]. Der Mini-TF-15 betont zusätzlich spezielle Hörbeeinträchtigungen durch den Tinnitus (Abb. 1, Faktor 2).

Vor dem Hintergrund verschiedener, nicht replizierter Ergebnisse zu möglichen Faktorstrukturen sollten derzeit lediglich die Gesamtscores der Minifragebögen verwendet werden.

Beide Fragebögen allein reichen nicht, um das komplexe Phänomen Tinnitus abzubilden [35, 36].

### Limitierungen

Die Berechnungen weisen zusätzlich zu den bereits erwähnten Einschränkungen weitere Limitierungen auf. So wurden die Items des Mini-TF-12 und Mini-TF-15 retrospektiv von einer umfassenden Stichprobe mit Ergebnissen der 52 Items umfassenden Originalversion des TF entnommen. Daher besteht die Möglichkeit abweichender Ergebnisse bei Verwendung des Mini-TF-12 und Mini-TF-15 im unmittelbaren Vergleich. Darüber hinaus wäre die Einbeziehung weiterer Variablen, wie Ergebnisse von Hörtests, Sozial- und Gesundheitsdaten sowie Daten zur Dauer des Tinnitus von großem Interesse und könnten zu einem besseren Verständnis tinnitusassoziierter Beschwerden und Einschränkungen beitragen. Des Weiteren erlaubt das retrospektive Studiendesign keine Aussagen über den Verlauf des Belastungserlebens bei betroffenen Patienten oder mögliche Unterschiede zwischen den Patientengruppen hinsichtlich der Ansprechrate auf verschiedene Therapieformen. Zum abschließenden Urteil der inhaltlichen Vor- und Nachteile der beiden Tests ist unbedingt noch eine Studie notwendig, die die beiden Kurzformen direkt miteinander vergleicht. Dies sollte effektiverweise im Rahmen von Interventionsstudien untersucht werden. Ergebnisse mit dem Mini-TF-12 zur Abbildung therapeutischer Effekte liegen vor, z. B. für die Stabilität einer Verbesserung nach einer Behandlung in einer Tinnitusspezialambulanz [30]. Studien mit dem Mini-TF-15 dazu stehen noch aus. Zudem sollten die beiden aus dem deutschen TF abgeleiteten Kurzformen auch in einem direkten Vergleich zu klinisch erprobten Kurzformen anderer internationaler Tinnitusfragebögen ihre Vorteile beweisen, z. B. der Kurzform des THI [2, 17].

Folgestudien sind notwendig, um die Reproduzierbarkeit der Ergebnisse und insbesondere psychometrische Eigenschaften des Mini-TF-15 in anderen Stichproben von Patienten mit chronischem Tinnitus weiter zu untersuchen. Dabei wäre auch der Einsatz zusätzlicher Variablen wie Sozialdaten, Ergebnisse von Hörtests sowie Tests zur Erfassung exekutiver Funktionen und Copingmechanismen von besonderem Interesse. So könnten mögliche Subgruppen von Patienten mit chronischem Tinnitus ermittelt werden, die in bestimmten Domänen eine besonders ausgeprägte Belastung zeigen und andersherum mögliche protektive Faktoren identifiziert werden.

## Ausblick und klinische Empfehlung

Die Behandlung des chronischen Tinnitus muss auf fundierter Diagnostik mit Erfassung von Tinnitusparametern inklusive audiologischer Komponenten (Tinnitusfrequenz und -lautstärke, Hörvermögen) sowie psychologischer Konstrukte basieren. Die Messung von Tinnitusbelastung sollte mittels validierter und international vergleichbarer Fragebögen erfolgen und unbedingt psychologische Erlebens- und Reizverarbeitungsdimensionen umfassen – und sich nicht auf Symptomchecklisten beschränken.

Zur Einordnung und Interpretation der Struktur der Kurzfragebögen sei auf aktuelle Studien verwiesen. Faktoranalytisch wurden als der Tinnitusbelastung zugrunde liegend 5 Merkmale bestimmt: „Stress“, „Schmerz“, „Erschöpfung“, „Kontrolle“ und „Bildungsniveau“ [8]. Ein anderes komplexes Modell [14] unterteilt den Tinnitus anatomisch und phänomenologisch in 3 Bahnen: eine laterale Bahn zur Verarbeitung des Klangs oder Höreindrucks, eine mediale Bahn, wo Leidensdruck entsteht und eine absteigende Geräuschunterdrückungs- oder Kontrollbahn. Diese Bahnen werden Netzwerken zugeordnet. Dabei soll es bei Tinnitus wie bei anderen neuropsychiatrischen Störungen zu abnormalen Interaktionen zwischen 3 kardinalen Hirnnetzwerken – dem selbstrepräsentativen Default-Mode-Netzwerk, dem verhaltensrelevanten, kodierenden Salienznetzwerk und dem zielorientierten zentralen Exekutivnetzwerk – kommen. Konkret führt Tinnitus damit in der Regel zu negativen kognitiven, emotionalen und autonomen Reaktionen, die sich phänomenologisch als tinnitusbedingtes Leiden ausdrücken und von der medialen Signalbahn verarbeitet werden sollen. Diese überschneidet sich anatomisch mit dem Salienznetzwerk, das die Verhaltensrelevanz des Schallreizes kodiert. Chronifizierung des Tinnitus kann auch mit dem selbstrepräsentativen Default-Mode-Netzwerk assoziiert werden und wird zu einem festen Bestandteil der Selbstwahrnehmung [14].

Der Mini-TF-12 scheint dabei besonders geeignet, psychosomatische Komorbidität, besonders Depression und dabei das Leiden an Tinnitus zu erfassen (mediale Bahn) [6, 31]. Der Mini-TF-15 scheint bezüglich der diskriminanten Validität von psychiatrischen Komorbiditäten etwas besser zu sein und erfasst zusätzlich Höreinschränkungen (laterale Bahn) und eventuell auch Geräuschunterdrückungsversuche (exekutives Netzwerk). Der Mini-TF-15 wurde allerdings bisher ausschließlich im deutschen Sprachraum angewendet und validiert, eine Überprüfung internationaler Vergleichbarkeit und seiner Eignung für die Abbildung von Therapieeffekten sollte perspektivisch erfolgen.

## Fazit für die Praxis

- Diese Reanalyse bezüglich der Itemcharakteristika der beiden Kurzfragebögen Mini-TF-12 und Mini-TF-15 zeigt eine gleichermaßen ausgeprägte Eignung zur Erfassung und zum Verständnis tinnitusassoziierter Beschwerden in verschiedenen Bereichen.
- Bei guter Reliabilität und akzeptabler Validität sind beide Selbstbeurteilungsinstrumente zur differenzialdiagnostischen Einordnung und Behandlungsplanung für betroffene Patienten und Behandler von großer Bedeutung.
- Beide Kurzversionen stellen eine gut validierte und auch bezüglich der Items reliable Kurzversion des TF dar, die zur schnellen, wirtschaftlichen und gleichzeitig differenzierten Erfassung tinnitusabhängiger Beschwerden zum Einsatz kommen kann.
- Damit können beide Kurzfragebögen zu einer individuelleren Therapieplanung und Erhöhung der Therapieresponse sowie zur Erfassung psychischer Belastung und Komorbidität bei Patienten mit chronischem Tinnitus beitragen.
